# Supplementary material for: Salt-Related Knowledge, Attitudes and Behaviors (KABs) among Victorian Adults Following 22-Months of a Consumer Awareness Campaign
Source: Nutrients. 2020 Apr 26;12(5):1216. doi: 10.3390/nu12051216 (PMC7282017; doi:10.3390/nu12051216)
Supplement: Supplementary file 1 [file nutrients-12-01216-s001.zip › supple-final/Grimes Supplementary File 1. Salt KAB questionnaire.pdf]

Victorian Survey – Knowledge, Attitudes and Behaviours related to Dietary Salt Intake

Table 1. Overview of questions included follow up KABs survey (T2 2018 & T3 2019): note 3 additional Qs have been added since completion of the 2015 baseline KABs survey. This includes 2 knowledge Qs and 1Q related to awareness of campaign, all are highlighted in red below.

**Question Type**	**Questions to include**	**No. of questions**
Demographic Questions	- Sex<br>- Age<br>- Country of birth<br>- Language<br>- Residential postcode<br>- Highest level of education attained<br>- CVD related medical conditions<br>- Medication for BP (if answered yes to high blood pressure)<br>- Dietary advice on limiting salt from health professional<br>- Weight<br>- Height<br>- Main shopper in household	11 (Qs 1-11)
Knowledge	- Q12 Relationship between salt and sodium<br>- Q13 Knowledge of how much salt Australians consume<br>- Q14 Main food source of salt in the diet<br>- Q15 Salt intake recommendations<br>- Q17 Is salt harmful to health<br>- Q18 Associated health risks<br>-Q19 Matrix question. Identification of food items containing added salt (only added at T2 & T3)<br>-Q20 NIP question. Lowest sodium content on bread label (only added at T2 & T3)	8
Attitudes	- Q16 Personal attitude to own salt intake<br>- Q21 Concern about salt and other food related issues<br>- Q22 Block question. Series of agree/disagree statements to attitudes related to salt intake (Note Q on Himalayan salt included as knowledge and in knowledge score)<br>- Q23 Responsibility for salt reduction	4
Behaviours	- Q24 Salt use at the table<br>- Q25 Salt use during cooking<br>- Q26 Salt shaker placed on table<br>- Q27 Trying to reduce salt in diet<br>- Q28 Block question, assessing salt related behaviours in previous month (e.g. reading labels, take-out foods, using herbs/spices)	5
Health star food label	- Q29 Question about use of health star label	1
Awareness of VicHealth salt initiative<br>Process evaluation Q	- Q30 Awareness of VicHealth salt reduction campaign<br>- Q30.1 How did you hear about the campaign and source used to view content of campaign	2
Parent	- Q31 One question to determine if participant is a parent or primary carer of child/children <18 years	1
**Total Questions**		**32**

Additional Questions for parents/caretakers	- Q32 Age of child/children<br>- Q33 Salt use in food prepared for child/children (behaviour)<br>- Q34 Salt shaker on table (behaviour)<br>- Q35 Child’s salt use at table (behaviour)<br>- Q36 Knowledge of how much salt Australian children eat (knowledge)<br>- Q37 Importance of limiting salt in their child’s diet (attitude)<br>- Q38 Knowledge of long term health effects in children (knowledge)<br>- Q39 Should more action be taken to reduce salt in foods targeted at children (attitude)	8
**Total Questions for parents**		**40**

* Note all questions have been set as forced entry. Some sensitive demographic questions, such as education level, have a ‘prefer not to answer’ response. For height and weight, there is a don’t know/prefer not to answer option for participants who are part of Facebook, consumer research panel or shopping centre survey – survey only sample. For shopping centre participants with a urine sample these questions are mandatory.

Victorian Survey – Knowledge, Attitudes and Behaviours related to Dietary Salt Intake

Thank you for taking the time to complete this survey. There are two sections to this survey. Please answer each question by selecting the most suitable option.

All responses on this survey will remain anonymous.

SECTION ONE – DEMOGRAPHIC INFORMATION

In this section we are interested in finding out a little bit of background information about you. This information will help us compare the answers of respondents from different backgrounds.

Q1. What is your sex? *Source: [1]*

Male
Female

Q2. What is your age in complete years? *Source: [1]*

____

Q3. In which country were you born? *Source: [2] (*Note VicHealth Indicators Survey as per Jane Shill. Also consistent with Victorian Population Health Survey)*

Australia (includes External Territories)
United Kingdom (incl. England, Scotland, Wales, Northern Ireland)
New Zealand
Italy
Greece
China
Vietnam
Lebanon
Other, please specify ______________________
Don't know
Prefer not to answer

Q4. Do you speak a language other than English at home?
*(If more than one language, indicate the one that is spoken most often) Source: [2]. (*Note VicHealth Indicators Survey as per Jane Shill. Also consistent with Victorian Population Health Survey)*

No, English only
Yes, Italian
Yes, Greek

Yes, Cantonese
Yes, Mandarin
Yes, Arabic
Yes, Vietnamese
Yes, German
Yes, Spanish
Yes, Tagalog (Filipino)
Yes, Other (please specify) ______________________
Don't know
Prefer not to answer

Q5. What is the postcode of your residential address?

____

Q6. What is the highest level of education and training you have completed? *Source: [2] (*Note VicHealth Indicators Survey as per Jane Shill. Also consistent with Victorian Population Health Survey)*

Never attended school
Some primary school
Completed primary school
Some high school (i.e Year 7 to Year 11, Form 1 to Form 5)
Completed high school (i.e. Year 12, Form 6, HSC)
TAFE or Trade Certificate or Diploma but did not complete Year 12 at secondary school
TAFE or Trade Certificate or Diploma and also completed Year 12 at secondary school.
University, or some other Tertiary Institute degree, including post university (i.e. postgraduate diploma, Master's degree, PhD)
Other, please specify _________________________
Don't know
Prefer not to answer

Q7. Have you ever been diagnosed with or suffered from one or more of the following conditions? *(You may select more than one)*

Heart disease

Stroke
High blood pressure
Heart attack
Other (please specify: ___________)
Can't recall/ Don't know
No

Q7.1 (if Yes to High blood pressure response)

Do you currently take medication for the control of your blood pressure? *Source: [3]*

Yes
No

Q8. Have you ever received any advice from your doctor or a health professional to reduce your intake of salt/sodium and/or salty foods? *Source: [3]*

Yes
No
Cant' recall

Q9. How tall are you without shoes?

You can enter this in cm or feet and inches. **Only fill in one option.** *Source: [1].*

_____________ Centimetres (e.g. 165)

OR

_____________ Feet and inches (e.g. 5 feet 4 inches)

Don't know/prefer not to answer

Q10. How much do you weigh without clothes and shoes?

You can enter this in kilograms OR pounds and stones. **Only fill in one option.***(Source: [1]*

______________ Kilograms (e.g. 74)

OR

______________Stones and pounds (e.g. 11st 10lbs)

Don't know/prefer not to answer

Q11. Are you the main person who does the grocery shopping in your household? *Source [4]*

Yes

No
I share the responsibility

SECTION TWO – YOUR VIEWS ON SALT INTAKE

[Questions related to knowledge]

In this section we are interested in finding out more about your views on dietary salt.

Q12. On Australian food products information about the amount of sodium within a food product is displayed on the food label. What is the relationship between salt and sodium? *Source: Modified [4-6]*

	**Knowledge Q**
1	They are exactly the same
2	**Salt contains sodium**
3	Sodium contains salt
4	I don't know/not sure

Q13. In general, how much salt do you think Australians eat? *Source: Modelled of [7, 8]*

	**Knowledge Q**
1	**Far too much**
2	**Too much**
3	Just the right amount
4	Too little
5	Far too little
5	Don't know

Q14. Which of the following do you think is the main source of salt in the Australian diet? *Source: Modelled of [5, 7]*

	**Knowledge Q**
1	Salt added during cooking or at the table
2	**Salt from processed foods such as breads, sausages and cheese**
3	Salt from natural food sources
4	Don't know

Q15. Health professionals recommend that we should eat no more than a certain amount of salt **each day**. How much salt do you think this is? *Source: Modelled of [4, 6, 9]*

	**Knowledge Q**
1	3 grams (about ½ a teaspoon)
2	**5 grams (about 1 teaspoon)**
3	8 grams (about 1 and a ½ teaspoons)
4	10 grams (about 2 teaspoons)
5	15 grams (about 3 teaspoons)
6	Don't know

Q16. How do you think your daily salt intake compares to the amount of salt recommended by health professionals? *Source: Modified [4, 5]*

I eat less salt than recommended
I eat about the right amount of salt
I eat more salt than recommended
I don't know

Q17. Do you think that eating too much salt could damage your health? *Source: [10, 11]*

	**Knowledge Q**
1	**Yes**
2	No
3	I don't know

Q18. Which, if any, of the following conditions do you think is linked to eating too much salt?

Please make sure you select an option for each line. *Source: Modelled of [4, 5, 8, 10]*

	**Condition**
1	**High blood pressure**
2	**Kidney disease**
3	**Heart disease/heart attack**
4	**Stroke**
5	**Stomach cancer**

19. Below is a list of everyday food products. For each, please indicate whether **you** think the food product has salt added to it. If you don't know or are not sure of the answer, please select the "don't know/not sure" option. Please select one option for each food. *(Source Sarmugam et al., 2014)* *(*Note This question added at time point 2 and 3 to fit the messages in the campaign, question modified to fit messages in the campaign, these are indicated in red)*

**Food item**	**Yes**	**No**	**Don't know / Not sure**
Ham			
Tomato sauce			
White rice (boiled)			
Teriyaki stir fry sauce			
Beef steak (uncooked)			
Mixed fresh vegetables			
Gravy mix			
Bread			
Sausages			
Corn Flakes			
Cheddar cheese			
Sausage roll			
Yoghurt			

20. The following two nutrition information panels are taken from food labels found on two different types of bread. Please select the option (A or B) with the **LOWEST** salt content. *(Source modified Grimes et al., 2009)* **OPTION A IS CORRECT ANSWER** *(*Note This question added at time point 2 and 3 to fit the messages in the campaign)*

☐ Option A ☐ Option B I don't know/not sure

Nutrition Information Panel

Servings Per Package: 9.0
Serving Size: 85g (2 slices)

	Quantity Per Serving	Quantity Per 100g
Energy	844kJ	993kJ
Protein	8.2g	9.6g
Fat		
Total	1.5g	1.8g
Saturated	0.2g	0.3g
Carbohydrate		
Total	37.2g	43.8g
Sugars	2.3g	2.7g
Fibre	2.5g	2.9g
Sodium	340mg	400mg

Nutrition Information Panel

Servings Per Package: 10.0
Serving Size: 66g (2 slices)

	Quantity Per Serving	Quantity Per 100g
Energy	698kJ	1057kJ
Protein	5.9g	9.0g
Fat		
Total	1.6g	2.4g
Saturated	0.3g	0.5g
Carbohydrate		
Total	30.1g	45.6g
Sugars	1.5g	2.2g
Fibre	3.4g	5.2g
Sodium	317mg	480mg

[Questions related to attitudes]

Q21. Please indicate on the scale below how concerned you are about each of the following food related issues?

Please make sure you select an option for each line. *Source: Modelled of [12, 13]*

	Not at all concerned	Not very concerned	Somewhat concerned	Very concerned	Extremely concerned
Healthy eating					
The amount of sugar in food					
The amount of salt in food					
The amount of fat in food					
The amount of saturated fat in food					
The amount of kilojoules/calories in food					

Q22. Please indicate on the scale below how much you agree or disagree with the following statements.

Please make sure you select an option for each line. *Source: Modified [4, 12]*

**Statement**	**Strongly disagree**	**Disagree**	**Neither agree nor disagree**	**Agree**	**Strongly agree**
Himalayan salt, pink salt, sea salt and gourmet salts are healthier than regular table salt (**Incorrect**) ***(*Note this is a knowledge question)***					
I believe salt needs to be added to food to make it tasty					
My health would improve if I reduced the amount of salt in my diet					
It is hard to understand sodium information displayed on food labels					
When eating out at restaurants/cafes/pubs, I find that lower salt options are not readily available or only in limited variety					
There should be laws which limit the amount of salt added to manufactured foods					

Q23. From the list below please rate how responsible you think each group is for reducing the amount of salt Australians eat.**Please make sure you select an option for each line.** *Source: Modified [14]*

**Group**	**Not at all responsible**	**Somewhat responsible**	**Responsible**	**Very responsible**	**Don't know**
Government					
Food manufacturers					
Business (e.g. supermarkets, local markets)					
Chefs preparing foods in restuarants/pubs/cafes					
Friends/family					
Yourself					
Fast food chains					

[Questions related to behaviours]

Q24. How often do you add salt to your food at the table? *Source: [8]*

Always
Often
Sometimes
Rarely
Never
Don't know

Q25. In the food you eat at home, how often is salt added during cooking? *Source: [8]*

Always
Often
Sometimes
Rarely
Never
Don't know

Q26. Do you place a salt shaker on your table at meal times? *Source: Modified [15]*

Always
Often
Sometimes
Rarely
Never
Don't know

Q27. Are you trying to cut down on the amount of salt you eat? *Source: Modified [6, 8, 11, 13]*

Yes
No
Don't know

Q28. Below are some common ways to lower the amount of salt in your diet. In the past <u>month</u>, to what extent have you personally done any of the following?

Please make sure you select an option for each line. *Source modelled from:[8, 12]*

	Never do this	Rarely do this	Sometimes do this	Often do this	Always do this	Does not apply to me
Looked at a food label to check the salt/sodium content of a food item						
Avoided eating packaged, ready-to-eat foods						
Used spices/herbs instead of salt when cooking						
Avoided eating food from fast food restaurants (e.g. McDonalds, KFC, Pizza Hut)						
Avoided eating food from an Asian style restaurant or takeaway store (e.g. Chinese, Thai, Indian)						
Purchased foods labelled "no added salt", "salt reduced" or "reduced sodium"						
When eating out, asked to have your meal prepared without salt						

Q29. On some food products a health star rating is displayed on the food label.

[insert image of health star rating label]

Do you use the health star rating information to choose any of the following products?

	Usually	Sometimes	Never	Don't know
Breakfast cereals				
Cheese				
Bread				

Q30. Are you aware of VicHealth's initiative to reduce salt intake within the Victorian population?

Yes
No
Don't know

Q30.1. *If yes, where did you see or hear about the initiative to reduce salt in Victoria? Please tick one or more, as appropriate:*

The Heart Foundation's Don't trust your taste buds Website
Heart Foundation's Unpack the Salt website
Women's magazine
Radio
Internet video advertisements
Social media advertisements
Smartphone/tablet app video advertisements
Poster
Health professionals
Other, please specify:________________

Q31. Are you a parent or caregiver for a child or children under the age of 18 years?

No
Yes, I'm a parent
Yes, I care for a child/children

(if no directed to end of survey)

ADDITIONAL QUESTIONS FOR PARENTS

You have indicated that you are a parent or carer of a child or children. Because we are interested in what parents think about salt in children's diets we would like to ask you 8 more questions. This will take about **5 minutes** to complete**.** We greatly appreciate your time.

Q32. How old is your child/children or the child/children that you care for?

You can select more than one age category if needed.

0-1 year
2-4 years
5-12 years
13-17 years

Q33. Do you add salt to foods you prepare for your child or children? *Source modified: [8]*

Always
Often
Sometimes
Rarely
Never
Not applicable, I do not prepare food for my child

Q34. Do you place a salt shaker on your table at meal times? *Source: Modified [15]*

Always
Often
Sometimes
Rarely
Never
Don't know

Q35. Does your child or children add salt to their food at the table? *Source: Modified: [8]*

Always
Often
Sometimes
Rarely

Never
Don’t know

Q36. In general how much salt do you think Australian children eat? *(Source: Modified to be child specific [7, 8]*

Far too much
Too much
Just the right amount
Too little
Far too little
Don’t know

Q37. Please indicate on the scale below how much you agree or disagree with the following statement:

“Limiting the amount of salt my child/or children eat/s is important to me.”

Strongly disagree
Disagree
Neither agree nor disagree
Agree
Strongly agree

Q38. Please indicate on the scale below how much you agree or disagree with the following statement:

"In the long term eating too much salt during childhood may have harmful effects on children's health." *Source: Modified [5]*

Strongly disagree
Disagree
Neither agree nor disagree
Agree
Strongly agree

Q39. Medical research suggests that most Australian children eat more salt than is good for them. Do you think more action needs to be taken to reduce the salt in foods targeted at children? *Source modified: [5]*

Strongly disagree
Disagree
Neither agree nor disagree
Agree
Strongly agree

You have completed the Survey! Please hit the submit button.

Thank you for your time.

References

1. Australian Institute of Health and Welfare. *National Health Data Dictionary version 16;* AIHW AG: Canberra, 2012.

2. VicHealth. VicHealth Indicators Survey 2011. Available online: https://www.vichealth.vic.gov.au/programs-and-projects/vichealth-indicators-survey-2011. (Accessed on 23 Sep 2016).

3. Booth, A. Implementation strategies and effectiveness of nutritional messages for improving health. Victoria: Deakin University; 2007.

4. Grimes, C. A., Riddell, L. J., Nowson, C. A. Consumer knowledge and attitudes to salt intake and labelled salt information. *Appetite* **2009**, *53*, 189-94.

5. Australian Division of World Action on Salt and Health. 2007 Survey of Australian Consumer Awareness and Practices Relating to Salt Report. Available online: http://www.awash.org.au/wp-content/uploads/2012/10/AWASH_ConsumerSurveyReport_2007_05_15.pdf. (Accessed on 26 May 2015).

6. New Zealand Food Safety Authority. Salt Consumer Survey. Available online: http://www.foodsafety.govt.nz/elibrary/industry/salt-survey.pdf. (Accessed on 26th May 2015).

7. Papadakis, S., Pipe, A. L., Moroz, I. A., Reid, R. D., Blanchard, C. M., Cote, D. F., et al. Knowledge, attitudes and behaviours related to dietary sodium among 35- to 50-year-old Ontario residents. *The Canadian journal of cardiology* **2010**, *26*, e164-9.

8. World Health Organization. WHO STEPS Instrument (Core and Expanded) v3.1. Available online: http://www.who.int/chp/steps/instrument/en/. (Accessed on 26th May 2015).

9. Sarmugam, R., Worsley, A., Flood, V. Development and validation of a salt knowledge questionnaire. *Public Health Nutr* **2014**, *17*, 1061-8.

10. Consensus Action on Salt and Health. Salt and Your Health. TNS Public Opinion Survey. Available online: http://www.actiononsalt.org.uk/Docs/33386.pdf. (Accessed on 5th Aug 2015).

11. World Health Organization, Pan American Health Organization. *Protocol for Population Level Sodium Determination in 24-hr Urine Samples;* WHO/PAHO Regional Expert Group for Cardiovascular Disease Prevention through Population-wide Dietary Salt Reduction: Canada, 2010.

12. Arcand, J., Mendoza, J., Qi, Y., Henson, S., Lou, W., L'Abbe, M. R. Results of a National Survey Examining Canadians' Concern, Actions, Barriers, and Support for Dietary Sodium Reduction Interventions. *Can J Cardiol* **2013**, *29*, 628-31.

13. Food Standards Agency, COI Communications. *Consumer Attitudes to Food Standards Wave 5 - 2004;* London, 2005.

14. Newson, R. S., Elmadfa, I., Biro, G., Cheng, Y., Prakash, V., Rust, P., et al. Barriers for progress in salt reduction in the general population. An international study. *Appetite* **2013**, *71*, 22-31.

15. Grimes, C. A., Baxter, J. R., Campbell, K. J., Riddell, L. J., Rigo, M., Liem, D. G., et al. Cross-Sectional Study of 24-Hour Urinary Electrolyte Excretion and Associated Health Outcomes in a Convenience Sample of Australian Primary Schoolchildren: The Salt and Other Nutrients in Children (SONIC) Study Protocol. *JMIR research protocols* **2015**, *4*, e7.
