# Supplementary material for: Salt-Related Knowledge, Attitudes and Behaviors (KABs) among Victorian Adults Following 22-Months of a Consumer Awareness Campaign
Source: Nutrients. 2020 Apr 26;12(5):1216. doi: 10.3390/nu12051216 (PMC7282017; doi:10.3390/nu12051216)
Supplement: Supplementary file 1 [file nutrients-12-01216-s001.zip › supple-final/Grimes Supplementary File 2. Additional data.docx]

**Supplementary table 1.** Demographic characteristics of sub-sample of parents/caregivers, 2015 (n=544) and 2018 (n=799)

| Characteristic | 2015 (T1) | | | 2018 (T2) | | | P-value^b^ |
| --- | --- | --- | --- | --- | --- | --- | --- |
|  | n (unweighted) | % or mean (weighted)^a^ | 95% CI | n (unweighted) | % or mean (weighted)^a^ | 95% CI |  |
| Gender |  |  |  |  |  |  |  |
| Male | 282 | 46.2% | (42.0, 50.5) | 342 | 42.2% | (38.7, 45.9) | 0.16 |
| Female | 272 | 53.8% | (49.5, 58.0) | 457 | 57.8% | (54.1, 61.3) |  |
| Age (years) (mean) |  | 40.0 | (38.1, 39.9) |  | 39.1 | (38.4, 39.8) |  |
| Age group |  |  |  |  |  |  |  |
| 18-24 y | 26 | 7.6% | (5.2, 11.1) | 33 | 5.5% | (3.9, 7.6) | 0.37 |
| 25-34 y | 144 | 26.0% | (22.5, 29.8 | 214 | 29.9% | (26.6, 33.4) |  |
| 35-44 y | 229 | 37.4% | (33.4, 41.5) | 303 | 34.7% | (31.5, 38.1) |  |
| 45-54 y | 129 | 23.6% | (20.2, 27.5) | 202 | 23.7% | (20.8, 26.9) |  |
| 55-65 y | 26 | 5.4% | (3.7, 7.9) | 47 | 6.2% | (4.7, 8.2) |  |
| Country of Birth |  |  |  |  |  |  |  |
| Australia | 467 | 39.1% | (38.4, 39.8) | 638 | 39.1% | (38.4, 39.8) | 0.12 |
| United Kingdom | 11 | 80.3% | (77.3, 83.0) | 24 | 80.3% | (77.3, 83.0) |  |
| New Zealand | 7 | 3.0% | (2.0, 4.5) | 11 | 3.0% | (2.0, 4.5) |  |
| Other | 61 | 1.1% | (0.6, 2.1) | 119 | 1.1% | (0.6, 2.1) |  |
| Don't know/prefer not to answer | 8 | 14.7% | (12.4, 17.5) | 7 | 14.7% | (12.4, 17.5) |  |
| Do you speak a language other than English at home? |  |  |  |  |  |  |  |
| Yes | 98 | 17.8% | (14.7, 21.3) | 164 | 21.6% | (18.7, 24.8) | 0.01 |
| No, English only | 451 | 81.4% | (77.8, 84.6) | 614 | 76.0% | (72.7, 78.9) |  |
| Don't know/prefer not to answer | 5 | 0.8% | (0.3, 1.9) | 21 | 2.4% | (1.6, 3.7) |  |
| SES based on highest level of education^c^ |  |  |  |  |  |  |  |
| High SES | 220 | 39.2% | (35.1, 43.5) | 371 | 45.7% | (42.2, 49.3) | 0.08 |
| Mid SES | 183 | 33.2% | (29.3, 37.3) | 235 | 29.9% | (26.7, 33.3) |  |
| Low SES | 145 | 27.6% | (23.9, 31.7) | 193 | 24.4% | (21.4, 27.6) |  |
| BMI (kg/m2) (mean)^d^ |  | 27.0 | (26.4, 27.6) |  | 27.2% | (26.7, 27.7) | 0.62 |
| Weight category^d^ |  |  |  |  |  |  |  |
| Underweight | 11 | 2.6% | (1.4, 4.8) | 2.6 | 2.2% | (1.2, 3.9) | 0.68 |
| Healthy weight | 196 | 40.2% | (35.8, 44.8) | 40.2 | 37.4% | (33.7, 41.2) |  |
| Overweight | 156 | 32.4% | (28.3, 36.9) | 32.4 | 35.7% | (32.1, 39.4) |  |
| Obese | 118 | 24.7% | (21.0, 28.9) | 24.7 | 24.7% | (21.6, 28.1) |  |
| Diagnosed with a chronic condition |  |  |  |  |  |  |  |
| Yes | 165 | 29.2% | (25.5, 33.2) | 193 | 24.2% | (21.2, 27.4) | 0.03 |
| No | 379 | 69.2% | (65.1, 73.0) | 600 | 75.1% | (71.8, 78.1) |  |
| Don't know/can't recall | 10 | 1.6% | (0.9, 3.1) | 6 | 0.7% | (0.3, 1.7) |  |
| Have you ever been diagnosed with or suffered from one or more of the following conditions? (yes) |  |  |  |  |  |  |  |
| Heart Disease | 35 | 6.1% | (4.3, 8.5) | 31 | 4.0% | (2.8, 5.7) | 0.09 |
| Stroke | 32 | 5.4% | (3.8, 7.7) | 15 | 2.2% | (1.3, 3.6) | <0.001 |
| Heart attack | 21 | 3.4% | (2.2, 5.2) | 17 | 2.4% | (1.5, 4.0) | 0.31 |
| Other (please specify) | 59 | 5.4% | (3.8, 7.7) | 42 | 5.1% | (3.7, 6.9) | 0.79 |
| Don't know/can't recall | 10 | 1.6% | (0.9, 3.0) | 6 | 7.5% | (3.3, 1.7) | 0.12 |
| High blood pressure | 128 | 22.0% | (18.7, 25.6) | 135 | 17.0% | (14.5, 19.9) | 0.03 |
| *If yes, do you currently take medication for the control of your blood pressure?* |  |  |  |  |  |  |  |
| Yes | 87 | 69.1% | (60.4, 76.7) | 97 | 71.8% | (63.1, 79.1) | 0.65 |
| No | 41 | 30.9% | (23.3, 40.0) | 38 | 28.2% | (20.9, 36.9) |  |
| Have you ever received any advice from your doctor or a health professional to reduce your intake of salt/sodium and/or salty foods? |  |  |  |  |  |  |  |
| Yes | 141 | 24.7 | (21.2, 28.6) | 182 | 23.7% | (20.8, 27.0) | 0.93 |
| No | 381 | 69.5 | (65.4, 73.3) | 568 | 70.4% | (67.0, 73.6) |  |
| Can't recall | 32 | 5.8 | (4.0, 8.2) | 49 | 5.9% | (4.4, 7.7) |  |
| Are you the main person who does the grocery shopping in your household? |  |  |  |  |  |  |  |
| Yes | 436 | 80.3 | (76.7, 83.4) | 588 | 75.1% | (71.9, 78.0) | 0.06 |
| No | 36 | 6.1 | (4.3, 8.5) | 52 | 6.5% | (4.9, 8.6) |  |
| No, I share the responsibility | 82 | 13.6 | (11.0, 16.7) | 159 | 18.4% | (15.8, 21.3) |  |

**^a^** Demographic characteristics weighted to represent Victorian population (Census 2016) for age and sex (1)

**^b^** P-value determined via Pearson’s chi-squared test

**^c^** T1 n=548 as participants who responded “don't know” or “prefer not to answer” for highest level of education were excluded

**^d^** T1 n=481 and T2 n=708 as participants who responded with missing data or “don't know” or “prefer not to answer” for either height or weight were excluded


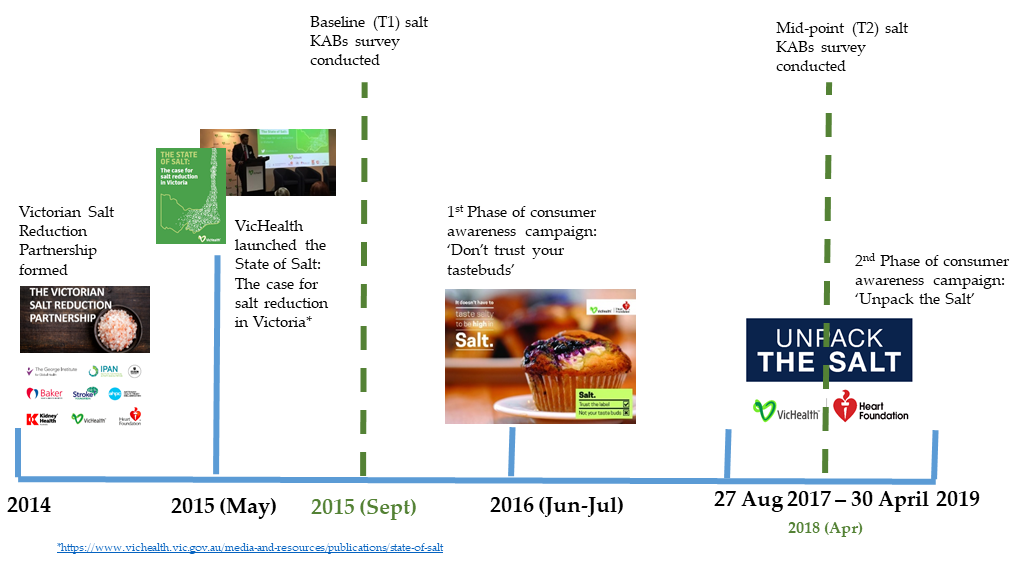


**Supplementary figure 1.** Timeline of salt reduction initiative and evaluation

**Supplementary figure 2.** Level of public concern for food-related issues among Victorian adults, 2015 (n=1584) and 2018 (n=2141)^a, b^

**^a^** Analysis weighted to represent Victorian population (Census 2016) for age and sex (31)

**^b^** Chi-squared test used to assess differences between T1 and T2. Healthy eating: P=0.73, amount of sugar in food: P=51, amount of salt in food: P=0.35, amount of fat in food: P=0.21, amount of saturated fat in food: P=0.06, amount of kilojoules/calories in food: P=0.19

**Supplementary figure 3.** Proportion of people who believe the following groups are responsible for reducing the amount of salt Australians eat, 2015 (n=1584) and 2018 (n=2141)^a, b^

**^a^** Analysis weighted to represent Victorian population (Census 2016) for age and sex (31)

**^b^** Chi-squared test used to assess differences between T1 and T2. Government: P=0.45, food manufacturers: P=0.33, Business (e.g. supermarkets, local markets): P=0.15, Chef’s preparing food in restaurants/pubs/cafes: P=0.27, friends/family: P=0.04, yourself: P=0.69, fast food chains: P=0.32
